# Use of Pharyngeal High-Resolution Manometry to Evaluate Dysphagia in Adults with Motor Neurone Disease: A Scoping Review

**DOI:** 10.1007/s00455-022-10418-4

**Published:** 2022-03-02

**Authors:** Eva Mary Diver, Julie Regan

**Affiliations:** Department of Clinical Speech and Language Studies, Trinity College Dublin, Dublin, Ireland

**Keywords:** Deglutition, Deglutition disorders, Pharyngeal high-resolution manometry, Motor neurone disease, Amyotrophic lateral sclerosis

## Abstract

There has been a recent shift towards proactive dysphagia intervention in motor neurone disease (MND) to maintain physiological reserve. Pharyngeal high-resolution manometry (PHRM) can quantify swallowing pathophysiology to inform and evaluate proactive dysphagia intervention. This study aims to explore the current use of PHRM as a dysphagia evaluation in adults with MND. A scoping review based on the Joanna Briggs Framework was completed. Four electronic databases (PubMed, EMBASE, CINAHL and Web of Science core) were searched (inception to March 2021) by two independent researchers. Data were analysed according to (i) PHRM protocol and analysis methods and the feasibility of same, (ii) swallow biomechanics data and (iii) dysphagia intervention effects as measured by PHRM. Six studies with 78 people with MND (PwMND) were included. There was considerable variation in PHRM protocol and analysis methods. Five studies reported a 100% completion rate and three studies reported no adverse events. Swallow biomechanics data were reported across all studies. The effects of sensory stimulation, increased bolus consistency, effortful swallow and cricopharyngeal myotomy were evaluated using PHRM with 20 PwMND across four studies with varying effects. Literature on the use of PHRM in PwMND is limited. Variability in PHRM methods restricts comparison of metrics. PHRM appears to be a feasible tool for PwMND. PHRM can provide novel swallow physiology data in PwMND and quantify discrete effects of compensatory and surgical dysphagia interventions not detectable by videofluoroscopy or FEES. Further research on the effects of proactive dysphagia intervention as measured by PHRM is required.

**Supplementary Information:**

The online version contains supplementary material available at 10.1007/s00455-022-10418-4.

## Introduction

Motor neurone disease (MND) is a life-limiting neurodegenerative condition characterised by the progressive decline of the motor neurones [1]. MND progresses rapidly, with average survival estimated at 2–4-year post-diagnosis [2]. The International Statistical Classification of Diseases and Related Health Problems (ICD-10) acknowledges four subtypes of MND [3]. Amyotrophic Lateral Sclerosis (ALS) is the most prevalent of the subtypes. The remaining subtypes include Progressive Bulbar Palsy, Progressive Muscular Atrophy and Primary Lateral Sclerosis [3]. At this point in time, there is no reversible treatment for what is typically a catastrophic collapse of a previously apparently normal functioning motor system. Symptomatic management is the main course of treatment [4]; thus, identification and exploration of mechanisms of impairment by researchers and clinicians are of significant importance.

One of the most frequent and devastating symptoms of MND is dysphagia, a group of symptoms characterised as difficulty forming the bolus and progressing it safely and efficiently from the mouth to the stomach [5]. Evidence suggests that almost all people with MND (PwMND), regardless of subtype, will eventually experience some degree of dysphagia [6]. Dysphagia in MND may occur during any stage of the swallow due to the weakening of the bulbar, respiratory and limb musculature [7, 8]. As MND progresses, individuals will experience a worsening of dysphagia symptoms, leading to complications, such as dehydration, weight loss, malnutrition and aspiration pneumonia [1, 8]. Indeed, aspiration pneumonia is the leading cause of death in this clinical population [9].

Traditionally, a compensatory approach has been taken to dysphagia management in the MND population. As part of this disease-centric reactive approach, the focus has been on palliative interventions [7]. In an effort to optimise physiological reserve for swallowing early in the disease process, there has been a recent shift towards proactive dysphagia management [10]. This approach involves proactively targeting underlying physiological function before the development of dysphagia to optimise physiological reserve. To accommodate for this shift in dysphagia management, an instrumental dysphagia evaluation which can capture discrete alterations to swallow physiology from early stages of the disease is needed to identify therapeutic targets in dysphagia treatment.

Pharyngeal high-resolution manometry (PHRM) is an emerging technology gaining increasing interest as a method for assessing pharyngeal swallow function [11]. In contrast to videofluoroscopy (VFS) and fiberoptic endoscopic evaluation of swallowing (FEES), which focus largely on swallow safety and efficiency, PHRM objectively identifies abnormalities in pharyngeal function through the quantification of pressure changes across the pharynx. To date, PHRM provides novel data on the velopharynx, mesopharynx, hypopharynx and the UES [12], all of which are affected in the MND population [1]. PHRM can clarify the biomechanical foundations of dysphagia that cannot be understood from visualisation alone, which would contribute to an enhanced, more targeted intervention plan for the PwMND. Whilst PHRM provides novel data on swallow physiology, within-subject pressure variability has been reported during volitional swallowing tasks [13]. The effect of age, pharyngeal region and volume on pharyngeal swallow pressure variability has also been established [14]. Furthermore, pressure drift has been reported across studies [15]. Each of these issues have the potential to impact markedly on clinical judgement and require further investigation before PHRM can be fully integrated into clinical practice.

Whilst PHRM would ensure the identification of subtle physiological changes of the swallow in PwMND, which, with the progressive nature of MND, could prove integral in preventing devastating dysphagia complications, there is a significant gap across the literature summarising the adoption of PHRM into dysphagia evaluation in PwMND. Such research is vital. PHRM is an invasive procedure and prevalent features of MND such as weak cough and hyperactive gag reflex could potentially impact patient acceptability and thus the overall feasibility of this assessment for PwMND [6, 7].

The primary aim of this study is to explore and summarise the use of PHRM to evaluate dysphagia in PwMND. In doing so, the following research questions will be addressed:

1. What PHRM protocol and analysis methods are currently being used with PwMND and are they feasible for this clinical population?
2. What are the swallow metrics obtained from PHRM in PwMND and how do these compare to metrics from healthy adults?
3. What are the effects of swallowing interventions in PwMND as measured by PHRM?

## Methods

### Methodological Approach

A scoping review was completed based on the methodological framework by the Joanna Briggs Institute (JBI) [16]. The JBI framework provides more explicit detail of the methodological steps than prior frameworks. This enhancement of detail increases the rigour and clarity of the review process and was thus selected for this study. The Preferred Reporting Items for Systematic Reviews and Meta-Analyses extension for Scoping Reviews (PRISMA-ScR) [17] was also used to highlight potential methodological issues and enhance the reporting quality of this paper (see appendices).

### Inclusion Criteria

1. Studies involving adults (> 18 years) that presented with any subtype of MND, under the ICD-10 code G12.2 [3], that underwent PHRM as an oropharyngeal swallow assessment or as a measure of intervention effects from which pressure metric data could be extracted were included in this study.
2. No constraints were applied regarding the geographical location of the study or the year of publication.
3. Abstracts of studies yet to be published were included provided sufficient data could be drawn.
4. Studies reporting data on a heterogeneous population that included some participants with MND were included.
5. Studies documenting metric data were included regardless of what oropharyngeal metric data they provided, so long as they reported on at least one UES or pharyngeal metric.
6. Papers that report the use of PHRM with or without impedance were included.
7. Records investigating the effects of any intervention type; rehabilitation, surgical, compensatory and/or environmental were included, provided adequate data were provided.

### Exclusion Criteria

1. Studies using water-perfused manometry, balloon-based manometry or 3D PHRM systems were excluded. Based on the PHRM international working group’s recommendations, studies that use solid-state manometry with less than ten pressure sensors more than 1 cm apart were excluded [18].
2. Review papers or papers presenting previously published data were excluded (e.g. [19]).
3. Studies that did not provide extractable quantitative metric data on the PwMND and whose authors could not provide such data or did not respond were excluded.
4. Studies published in any language other than English were excluded.

### Search Strategy

A comprehensive search string was developed by the researcher and peer-reviewed by a qualified university research librarian using the PRESS-EBC Checklist [20]. All included search strings are listed in the appendices section. The search strategy applied in PubMed is reported as an example (performed on 10/03/2021) deglutition*[Title/Abstract] (T/A) OR swallow* (T/A) OR oropharyngeal (T/A)OR pharyn*(T/A) OR dysphagi*(T/A) OR feed*[(T/A) OR fed (T/A) OR eat (T/A) OR eating (T/A) OR eats (T/A) OR ate (T/A) OR drink*(T/A) OR drank(T/A) OR deglutition[MeSH Terms] OR deglutition disorder[MeSH Terms]) AND (mixed etiolog*(T/A) OR mixed aetiolog*(T/A) OR motor neurone disease*(T/A) OR motor neuron disease*(T/A) OR MND (T/A) OR ALS (T/A)OR motor system disease*(T/A) OR anterior horn cell disease*(T/A)OR lateral sclerosis (T/A) OR lateral scleroses (T/A)OR progressive bulbar palsy (T/A)OR bulbar paralysis(T/A)OR progressive muscular atroph*(T/A) OR charcot disease*(T/A) OR Lou Gehrig (T/A)OR motor neuron disease[MeSH Terms]) AND (manometry (T/A)OR high resolution(T/A)OR pharyngeal pressure*(T/A) OR manometry[MeSH Terms]).

## Procedure

### Information Sources

Four electronic databases were searched from the inception of the database to the 10^TH^ of March 2021, including PubMed, EMBASE, CINAHL, and Web of Science Core. No limits, i.e. language or date, were put on the search. The Dysphagia Journal and the Amyotrophic Lateral Sclerosis Journal, as well as the 2020 published conference abstracts of the Dysphagia Research Society (DRS) (available via DRS website) and the European Society of Swallowing Disorders (ESSD) (available via the ESSD website) were reviewed. The bibliography of all the identified publications screened by the titles and citations were tracked via the Google Scholar website. All searches were conducted by the primary researcher (ED).

### Screening the Evidence

All retrieved citations were imported into EndNoteX9 software [21] for data management and storage. Citations were exported to Covidence [22] for removal of duplicates and screening. Title and abstracts and full texts were screened by two independent researchers (ED) and (JR). A full 100% agreement level was achieved. The final data extraction was performed by an independent researcher (ED).

### Data Synthesis

Due to the heterogeneity in PHRM protocol and equipment and the ability for technique or catheter configuration to impact results, this review concentrated on regional changes in relation to swallowing outcomes. This knowledge should be considered when interpreting the metric data and as such, rather than conducting a statistical synthesis, a narrative analysis was performed.

PHRM protocol and analysis methods, metric results of PwMND and intervention effects were tabulated, interpreted and given meaning through discussion and description in the narrative texts. Data regarding the feasibility of the assessment were also analysed and descriptively discussed.

The metric results of PwMND were analysed and compared against the normative data outlined in that same study; this was to establish consistency across PHRM protocol and analysis methods and thus ensure true comparability of results. The comparisons were translated into a bar chart graphic to enable visualisation and further exploration of the changes in the swallow in PwMND.

## Results

### Selection of Sources of Evidence

**Fig. 1:**
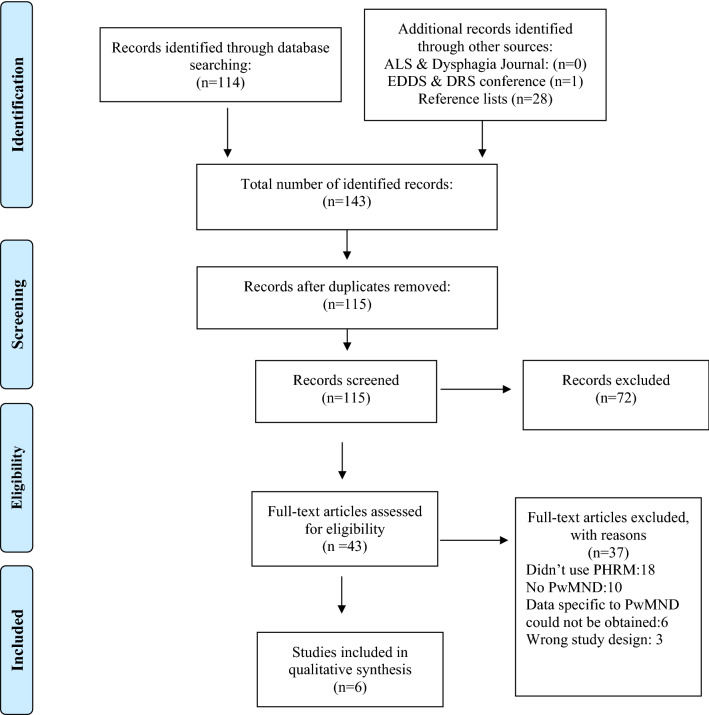
Prisma 2009 flow diagram

A total of 143 studies were identified from the search across the aforementioned databases, journals, conference abstracts and reference lists. After deduplication, 115 studies remained. A total of 72 studies were excluded after title and abstract screening, leaving 43 studies for full-text review. Eventually, six studies, one of which was an abstract of a study yet to be published, remained and were included in this review. Figure 1 illustrates the PRISMA flow diagram of study selection.

Of the six studies included, 1 was a single-case study [23], 2 were case–control study designs [24, 25] and 3 were quasi-experimental designs (pre-test–post-test designs) [26–28]

### Participant Demographics

There was considerable variation concerning sample size across studies; on average, studies included 11 participants with MND. Male patients constituted 54% of patients across studies; this accurately represents the Male:Female predominance in MND [29]. The average age of patients across studies was 68 ± 10 years; this too is representative of the MND population as the peak incidence of MND falls between 60 and 75 years [30]. Documentation on dysphagia severity varied across studies. 50% of studies outlined the participants functional oral intake scale (FOIS) severity, FOIS 1 being nil by mouth and FOIS 7 being total oral diet. Further information regarding patient demographics is outlined in Table 1.

**Table 1:** Participant demographics

Study	Number Pts	Gender	Age	MND classification	MND severity	Time since MND diagnosis	Dysphagia severity
Takasaki et al. 2010	1	Male	60 yrs	NR	NR	1.5 yrs	Descriptive: presented with intractable aspiration
Regan, 2020	2	1 Male 1 Female	82 yrs 78 yrs	Bulbar onset	NR	NR	FOIS score: 4 FOIS score: 3
Heslin, 2020	2	1 Male 1 Female	82 yrs 78 yrs	Bulbar onset	NR	NR	FOIS score:4 FOIS score:3
Suh et al., 2019	41	21 Male 20 Female	65 ± 11 yrs	NR	NR	NR	FOIS 1 group FOIS 2/3group FOIS4/7 group (Demographics of groups NR)
Cock et al., 2019	16	8 Male 8 Female	70 ± 8 yrs	Bulbar group Pseudobulbar group (Demographics of groups NR)	NR (Abstract)	NR (Abstract)	NR (Abstract)
Cock et al., 2015	16	10 Male 6 Female	70 ± 9 yrs	Lower MN involvement (11pts) Upper MN involvement (5pts)	NR	NR	Descriptive: All Pts had moderate- severe dysphagia; none were tube fed

*FOIS*, functional oral intake scale; *MN*, motor neurone; NR, not reported; Pts, participants; yrs, year

### PHRM Protocol and Analysis Methods & the Feasibility of Such Methods for PwMND

All six studies are included in the PHRM equipment and data acquisition Sect. 5 studies are included in the feasibility section.

### PHRM Equipment

The majority of studies did not provide full documentation of the PHRM equipment used. Variability in the type of PHRM equipment used was noted across studies that did report such information. Of the studies that documented PHRM system, the Mano scan was most reported (50%) followed by the MMS solar (33.3%) and Insight (16.6%) systems. Further information on PHRM equipment reported across studies is outlined in Table 2.

**Table 2:** PHRM protocol and analysis methods

Study	PHRM equipment				Data acquisition								Data analysis
	PHRM System	No of Pressure/ Impedance sensors; spacing in cm	Catheter diameter	Catheter direction	Fasting	Pt position	Topical Nasal Anaesthetic	Adjustment period	Bolus delivery method	No of trials	Bolus volume	Bolus consistency	Analysis software
Takasaki et al., 2010	Mano Scan	36 pressure sensors; 1 cm	4.2 mm	Circumference	NR	Supine	Yes	NR	NR	3	Dry swallow	Dry swallows	Mano-View
Regan, 2020	Mano scan	36 pressure sensors; 1 cm	4.2 mm	Circumference	4 h	Upright	No	5 min	Syringe	2	10 ml	Liquid	Swallow Gateway
Heslin, 2020	Mano scan	36 pressure sensors; 1 cm	4.2 mm	Circumference	4 h	Upright	No	5 min	Syringe	2	10 ml	Liquid	Swallow Gateway
Suh et al., 2019	InSight	36 pressure sensors; 1 cm but 2 cm in 5 places	NR	NR	Food-4 h Drink-2 h	Neutral head position	Yes	5–10 min	NR	2	5 ml	Water	Bio View Analysis
Cock et al., 2019	MMS solar	36 Pressure sensors; 1 cm 16 impedance sensors; 2 cm	NR (Abstract)	NR (Abstract)	NR (Abstract)	NR (Abstract)	NR (Abstract)	NR (Abstract)	NR (Abstract)	NR (Abstract)	5 ml	Normal Saline	MATLAB Algorithm
Cock et al., 2015	MMS solar	36 pressure sensors;1 cm & 16 impedance sensors; 2 cm OR 25 pressure sensors;1 cm & 12 impedance sensors; 2 cm	NR	Unidirectional	NR	Upright	Yes	15 min	Syringe	5	5 ml	Normal Saline	MATLAB algorithm

*Cm*, centimetre; *No*, number; *mm*, millimetre; *ml*, millilitre; *NR*, not reported; *Pt*, participant

### Data Acquisition

Variability in the protocol of data acquisition and documentation of this protocol across studies was also noted. 42.9% of studies reported the use of Topical Nasal Anaesthetic (TNA). The type of TNA was documented in 66.6% of these studies. Type ranged from lidocaine spray in one study [24] to co-phenylalanine forte spray and lignocaine gel in another [25]. 57% of studies used a 5 ml bolus volume, 28% used 10 ml, and 14% used dry swallows. The variability in the protocol of data acquisition is further outlined in Table 2.

### Data Analysis

The Analysis software used across studies is outlined in Table 2. One study [23] utilised the ManoView software. This software requires correction of a system-based measurement fault [31]. The study did not state whether the correction was made.

### Analysis of the Pharynx

**Fig. 2:**
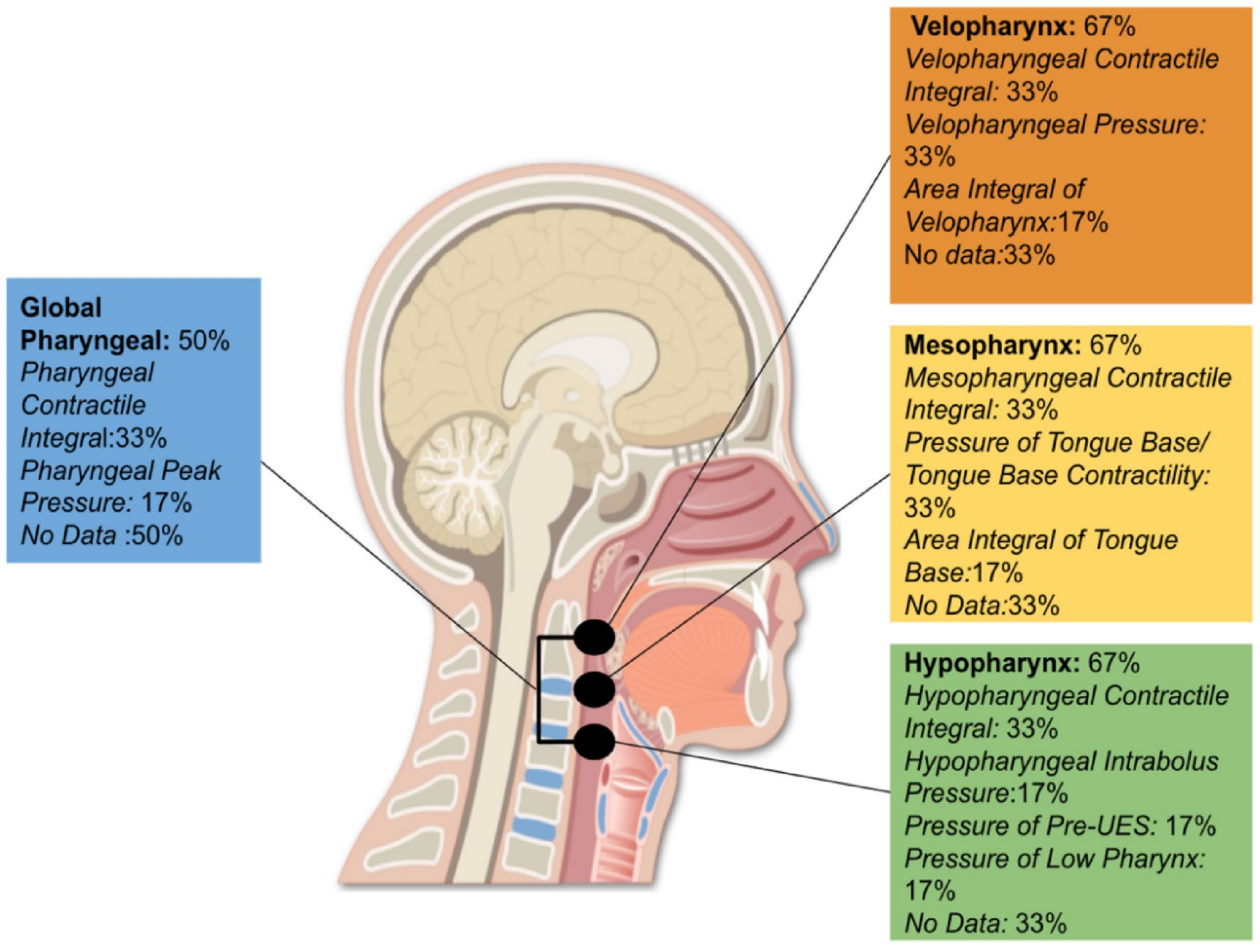
Pharyngeal metric sites and measurement parameters reported across studies and percentage of studies documenting each

Each of the six studies documented at least one pharyngeal measurement. Figure 2 illustrates the different pharyngeal metric sites and measurement parameters documented across the studies and the percentage of studies that reported each. The highest number of studies to report on the same exact parameter, i.e. velopharyngeal contractile integral, was 33.3% (two studies).

### Analysis of the UES

**Fig. 3:**
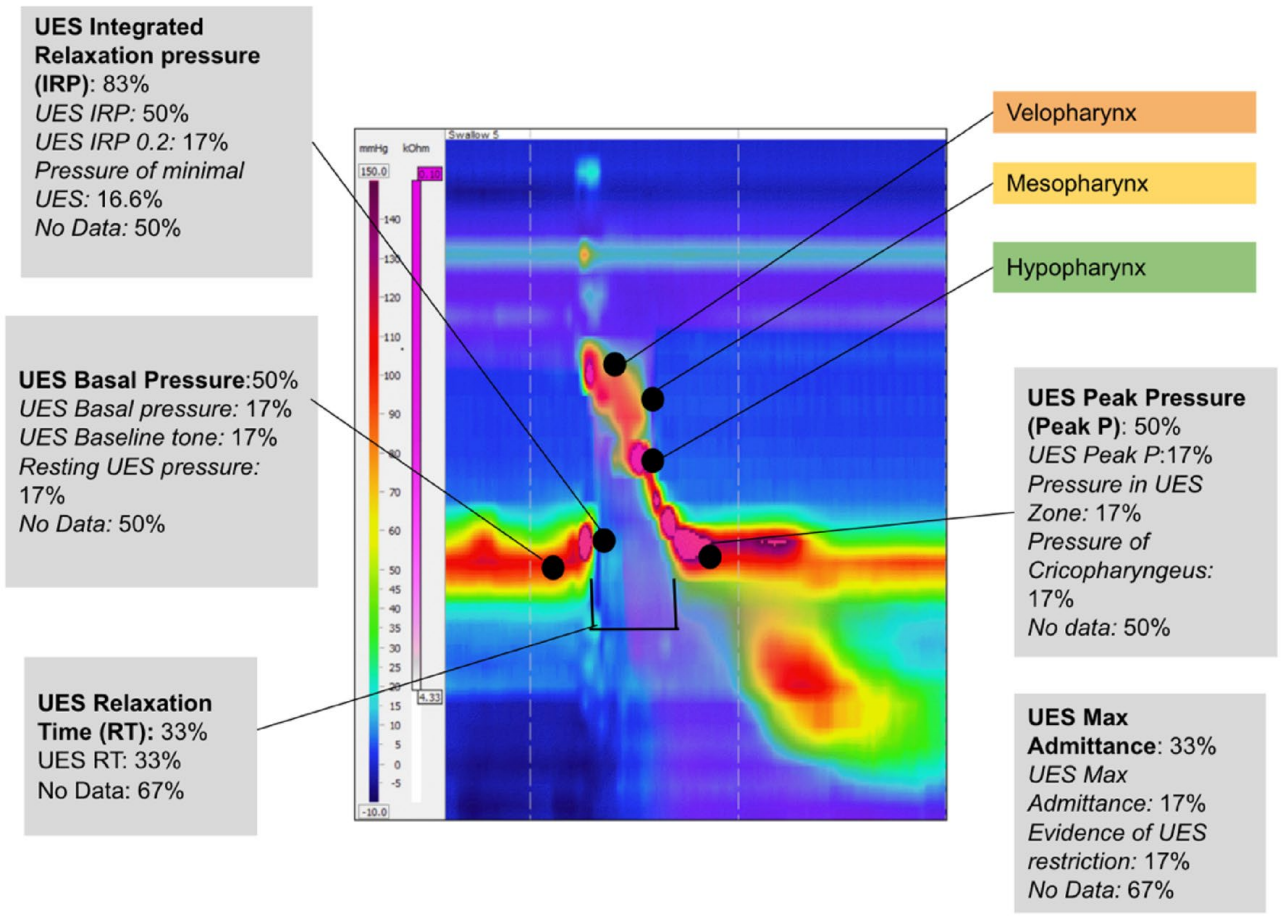
Upper oesophageal sphincter (UES) measurement parameters reported across studies and percentage of studies documenting each

Each study reported on the UES. The UES measurement parameters documented across studies and the percentage of studies that reported each is presented in Fig. 3. The number of studies reporting on the same measurement parameter of the UES (83.3%) was greater than that of the pharyngeal measures; however, differing definitions of this UES parameter (UES Integrated relaxation pressure) was noted across studies.

### Feasibility

One study [28], an abstract only, did not report PHRM outcomes. All of the remaining five studies documented a 100% completion rate of PHRM in participants with MND. Adverse events were reported in 60% of studies; 100% of these studies reported that no side effects or adverse events occurred as a result of PHRM. One study documented patient tolerability reporting that the patients tolerated the assessment well. None of the included studies documented participant-reported outcomes of the assessment.

### Swallow Biomechanics

#### Biomechanics of the Swallow in PwMND

Six studies are included in this section. A comprehensive overview of the PHRM metric results of PwMND reported across these studies is provided in Table 3.

**Table 3:** PHRM metric results for PwMND and healthy participants

Study	Metric	Definition of Metric reported	Sub-group of PwMND	Result	Normative data	Statistical Values
Velopharynx						
Suh et al., 2019	Velopharyngeal pressure (mmHg)	NR	FOIS1: FOIS2/3: FOIS4/7:	137 ± 34.31 146.13 ± 35.75 213.46 ± 62.29	208.88 ± 94.4	NR
Takasaki et al., 2010	Dry pressure in velopharyngeal Muscle Zone (mmHg)	NR	1Pt	95	141.1 ± 73.5	Maximum value
Regan, 2020	Velopharyngeal contractile integral (mmHg.cm.s)	Measure of contractile vigour within the velopharyngeal region only	FOIS3: FOIS4:	58.84 ± 13.97 36.155 ± 2.57	NR	Mean & Standard deviation
Heslin, 2020	Velopharyngeal Contractile integral (mmHg.cm.s)	Measure of contractile vigour within the velopharyngeal region only	FOIS 3: FOIS4:	14.9 14.91	NR	Median
Suh et al., 2019	Area integral of Velopharynx (mmHg.s)	NR	FOIS1: FOIS2/3: FOIS4/7:	35.5 ± 19.10 39.30 ± 35.01 52.30 ± 26.60	54.99 ± 35.37	NR
Mesopharynx						
Suh et al., 2019	Pressure of tongue base (mmHg)	NR	FOIS 1: FOIS2/3: FOIS4/7:	101.09 ± 20.24 99.10 ± 58.9 120.14 ± 31.00	144.4 ± 28.6	NR
Cock et al., 2019	Tongue Base contractility (mmHg)	NR	Pseudobulbar:	81 ± 14	151 ± 17	NR (Abstract)
Regan, 2020	Mesopharyngeal contractile integral (mmHg.cm.s)	Measure of contractile vigour within mesopharyngeal region only	FOIS 3: FOIS4:	29.565 ± 7.52 84.84 ± 23.48	NR	Mean & Standard deviation
Heslin, 2020	Mesopharyngeal contractile integral (mmHg.cm.s)	Measure of contractile vigour within mesopharyngeal region only	FOIS 3: FOIS4:	39.86 37.52	NR	Median
Suh et al., 2019	Area integral of tongue base (mmHg.s)	NR	FOIS 1: FOIS2/3: FOIS4/7	45.70 ± 12.30 45.85 ± 33.28 48.56 ± 24.20	54.67 ± 18.65	NR
Hypopharynx						
Cock et al., 2015	Hypopharyngeal intrabolus pressure (mmHg)	NR	MND Group	13 (7.6;21.5)	Aged controls: 8.9 (4.2;17.9) Young controls: 8 (3.4;13.6)	Median & Interquartile ranges
Suh et al., 2019	Pressure of low pharynx (mmHg)	NR	FOIS1: FOIS2/3: FOIS4/7:	177.01 ± 97.69 280.45 ± 98.03 351.89 ± 174.74	372.8 ± 164.1	NR
Suh et al., 2019	Pressure of Pre-UES (mmHg)	NR	FOIS1: FOIS2/3: FOIS4/7:	123.03 ± 59.9 140.29 ± 82.40 149.41 ± 57.52	194.96 ± 99.1	NR
Regan, 2020	Hypopharyngeal contractile integral (mmHg.cm.s)	Measure of contractile vigour within hypopharyngeal region only	FOIS3: FOIS4:	103.245 ± 35.67 99.73 ± 25.72	NR	Mean & Standard deviation
Heslin, 2020	Hypopharyngeal contractile integral (mmHg.cm.s)	Measure of contractile vigour within hypopharyngeal region only	FOIS 3: FOIS4:	110.02 61.76	NR	Median
Global pharyngeal measures						
Cock et al., 2015	Pharyngeal Peak Pressure (mmHg)	NR	MND Group:	77 (57;118)	Aged controls: 161 (117;221) Young controls: 136 (104;208)	Median & Interquartile ranges
Regan, 2020	Pharyngeal Contractile Integral (mmHg.cm.s)	Sum of pharyngeal pressure > 20 mmHg from superior pharyngeal constrictor margin to UES proximal margin over the period from UES opening to 0.5 s after UES closure	FOIS3: FOIS4:	125.56 ± 1.63 105.92 ± 9.12	NR	Mean & Standard deviation
Heslin, 2020	Pharyngeal Contractile Integral (mmHg.cm.s)	Sum of pharyngeal pressures > 20 mmHg from the velopharynx to the UES proximal margin over the period from UES opening to 0.5 s after UES closure	FOIS3: FOIS4:	164.78 114.19	NR	Median
UES						
UES relaxation time						
Regan, 2020	UES Relaxation Time (s)	A measure of duration of pressure drop at UES 50% below baseline or 35 mmHg	FOIS 3: FOIS4:	0.88 ± 0.001 0.485 ± 0.1	NR	Mean & Standard deviation
Heslin, 2020	UES Relaxation Time (s)	A measure of duration of pressure drop at UES 50% below baseline or 35 mmHg	FOIS3: FOIS4:	0.6 0.68	NR	Median
UES integrated relaxation pressure						
Regan, 2020	UES integrated relaxation pressure (mmHg)	A measure of the extent of UES relaxation – median of the lowest non-consecutive 0.20 – 0.25 s of pressure	FOIS3: FOIS4:	12.19 ± 0.13 0.235 ± 6.89	NR	Mean & Standard deviation
Heslin, 2020	UES integrated relaxation pressure (mmHg)	A measure of the extent of UES relaxation – median of the lowest non-consecutive 0.20 – 0.25 s of pressure	FOIS3: FOIS4:	6.49 4.8	NR	Median
Cock et al., 2019	UES integrated relaxation pressure (mmHg)	NR (Abstract)	Pseudobulbar:	6.1 ± 2.7	0.3 ± 1.1	NR
Cock et al., 2015	UES integrated relaxation pressure 2.0 (mmHg)	Median of the lowest pressures recorded over 0.2 cumulative, but not necessarily consecutive seconds	MND Group:	3.6 (0.7;6,9)	Aged controls: 3.6 (−0.2;8.7) Young controls: −1.6 (−3;2.3)	Median & Interquartile ranges
Suh et al., 2019	Pressure of minimal UES (mmHg)	NR	FOIS1: FOIS2/3: FOIS4/7:	1.65 ± 15.01 −7.33 ± 5.47 −10.02 ± 4.37	−7.97 ± 5.64	NR
UES maximum admittance						
Cock et al., 2015	UES max admittance (mS)	Highest level of UES admittance reached during relaxation	MND Group	2.7 (2.5;3.4)	Aged controls: 4.3 (3.5;5.6) Young controls: 5.6 (4.7;6.3)	Median & Interquartile range
Cock et al., 2019	‘Evidence UES restriction’(mS)	NR (Abstract)	Bulbar: Pseudobulbar:	3.7 ± 0.4 4.1 ± 0.3	7 ± 0.5	NR
UES basal pressure						
Regan, 2020	UES basal pressure (mmHg)	Pre-swallow basal pressure in UES defined as average UES profile pressure recorded over the period from 1 to 0.25 s prior to UES opening	FOIS3: FOIS4:	61.02 ± 1.05 29.79 ± 10.35	NR	Mean & Standard deviation
Cock et al., 2019	UES baseline tone (mmHg)	NR (Abstract)	Bulbar: Pseudobulbar:	12 ± 4 35 ± 5	55 ± 12	NR
Takasaki et al., 2010	Resting UES pressure (mmHg)	NR	1Pt	89	70.2 ± 30.0	Maximum
UES peak pressure						
Regan, 2020	UES peak pressure (mmHg)	UES post-relaxation peak pressure defined as maximum UES profile pressure recorded from 0 to 1 s after UES closure	FOIS3: FOIS4:	222.145 ± 2.8 280.36 ± 19.79	NR	Mean & Standard deviation
Takasaki et al., 2010	UES Zone (mmHg)	NR	1Pt	171	172.7 ± 73.8	Maximum
Suh et al., 2019	Pressure of cricopharyngeus (mmHg)	NR	FOIS 1: FOIS2/3: FOIS4/7	181.4 ± 107.91 200.90 ± 89.95 247.52 ± 78.85	388.2 ± 137.21	NR

*Cm*, centimetre; *FOIS*, functional oral intake scale; *mmHg*, unit of pressure; *NR*, not reported; *Pt*, participant; *s*, second

### Biomechanics of the Swallow in PwMND Compared to Normative Data

As data on a healthy control group was not included in 2 of the studies [26, 27], four studies are included in this section. Takasaki and colleagues [23] included retrospective normative data established in their previous study; the remaining papers established the normative data in the study included. All studies including normative PHRM metric data used the same PHRM protocol and analysis methods with the healthy participants and the PwMND.

**Fig. 4:**
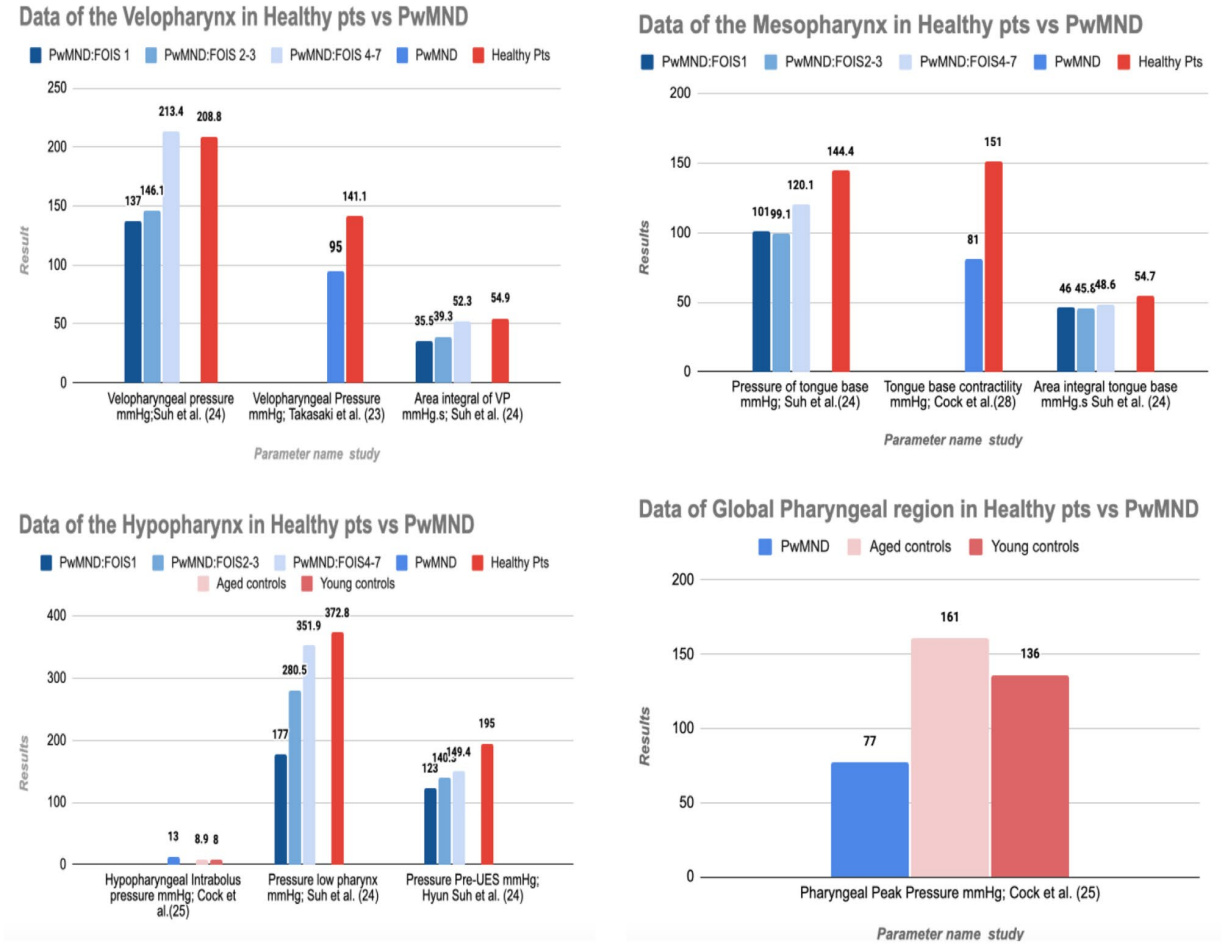
Pharyngeal metric data in healthy participants (red shades) vs PwMND (blue shades). PwMND and healthy participants are sub-grouped as per study, e.g. FOIS 1, FOIS2/3 and FOIS4/7

**Fig. 5:**
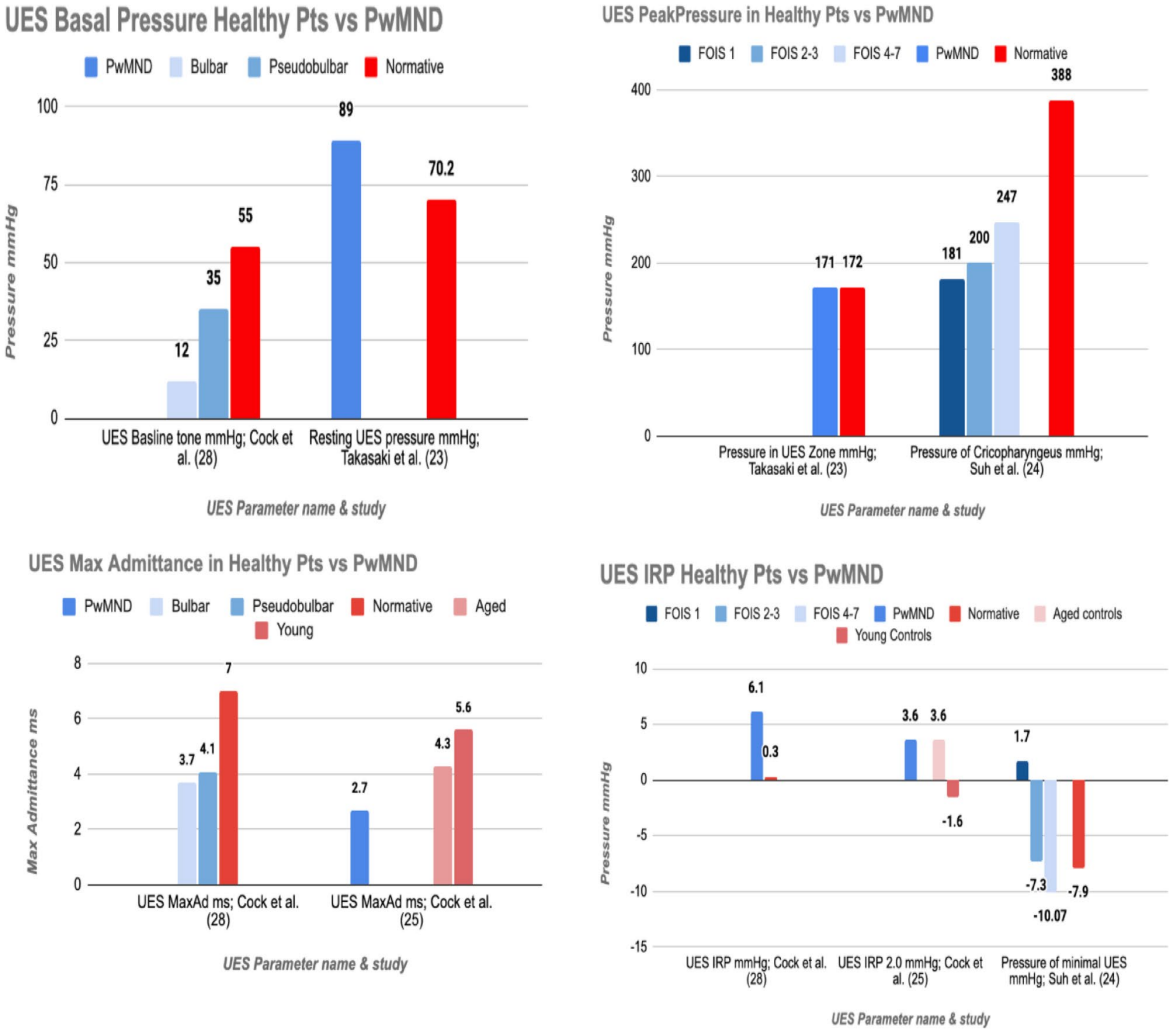
UES Metric data in Healthy Participants (shades of red) vs PwMND (shades of blue). Similarly as seen in Fig. 4 PwMND and healthy participants are sub-grouped as per study

Figures 4 and 5 outline the comparison between PHRM metric results in PwMND and healthy participants. Further detail on the metric results of the healthy participants is presented in Table 3.

### Intervention Effects

The effects of interventions in PwMND as measured by PHRM are discussed under the following four intervention categories: (i) Change to bolus characteristics, (ii) swallowing manoeuvres, (iii) surgical interventions and (iv) behavioural interventions. Four studies documented intervention effects and are thus included in this section. Table 4 outlines the effects of the various interventions.

**Table 4:** Effects of intervention

Study	Intervention	Intervention description	Metric	Baseline	Post intervention
Alteration in bolus characteristics					
Sensory stimulation					
				Neutral bolus	Bolus with Sensory Stimulation
Regan, 2020	Sensory stimulation	Duplicate 10 ml neutral (still water), sour (Lemon juice), cold (still water 3–5 degrees) and carbonated (Sparkling liquid) swallows given to participants in randomised order	Velopharyngeal Contractile Integral (mmHg.cm.s)	58.84 ± 13.97	Cold: 96.88 ± 10.73 Sour: 93.895 ± 0.46 Carbon: 107.69 ± 5.08
			Mesopharyngeal Contractile Integral (mmHg.cm.s)	29.565 ± 7.52	Cold: 43.685 ± 9.37 Sour: 75.66 ± 43.47 Carbon: 127.83 ± 3.6
			Hypopharyngeal Contractile Integral (mmHg.cm.s)	103.245 ± 35.67	Cold: 101.88 ± 18.63 Sour: 104.57 ± 30.14 Carbon: 134.92 ± 55.18
			Pharyngeal Contractile Integral (mmHg.cm.s)	125.56 ± 1.63	Cold: 187.61 ± 10.52 Sour: 189.7 ± 7.92 Carbon: 213.915 ± 49.5
			UES relaxation time (s)	0.88 ± 0.001	Cold: 0.67 ± 0.08 Sour: 0.875 ± 0.39 Carbon: 0.785 ± 0.16
			UES integrated relaxation pressure (mmHg)	12.19 ± 0.13	Cold: 6.17 ± 10.52 Sour: 14.665 ± 2.51 Carbon: 5.445 ± 2.65
			UES basal pressure (mmHg)	61.02 ± 1.05	Cold: 48.78 ± 6.38 Sour: 60.69 ± 0.06 Carbon: 84.645 ± 50.79
			UES peak pressure (mmHg)	222.145 ± 2.8	Cold: 127.385 ± 19.37 Sour: 262.155 ± 102.06 Carbon: 171.285 ± 38.49
Change in bolus consistency					
				Liquid bolus	Viscous bolus
Cock et al., 2015	Altering bolus consistency	Participants given 5 ml liquid (0.9% normal saline) and 5 ml Viscous bolus (Viscous Swallow Challenge Medium)	Pharyngeal peak pressure (mmHg)	77 (57;118)	69 (64;109)
			Hypopharyngeal intrabolus pressure (mmHg)	13 (7.6;21.5)	18.7 (12.3;24.1)
			UES-Integrated Relaxation Pressure 0.2 (mmHg)	3.6 (0.7;6.9)	6.9 (3.8;13.6)
			UES Maximum Admittance (mS)	2.7 (2.5;3.4)	2.9 (2.3;3.3)
Swallowing manoeuvres					
Effortful swallow					
				Normal swallow	Effortful swallow
Heslin, 2020	Effortful swallow	Participants complete a normal swallow and then told to swallow and “Squeeze hard with all of your muscles”	Velopharyngeal Contractile Integral (mmHg.cm.s)	P1: 14.9 P2: 14.91	9.5 59.89
			Mesopharyngeal Contractile Integral (mmHg.cm.s)	P1: 39.86 P2: 37.52	27.48 43.34
			Hypopharyngeal Contractile Integral (mmHg.cm.s)	P1: 110.02 P2: 61.76	59.19 38.77
			Pharyngeal Contractile Integral (mmHg.cm.s)	P1: 164.78 P2: 114.19	75.75 162.43
			UES Relaxation Time (s)	P1: 0.6 P2: 0.68	1.01 0.8
			UES-Integrated Relaxation Pressure (mmHg)	P1: 6.49 P2: 4.8	0.37 12
Surgical					
Cricopharyngeal myotomy					
				Prior myotomy	Post myotomy
Takasaki et al., 2010	Cricopharyngeal myotomy	Bilateral cricopharyngeal myotomy	Resting UES pressure (mmHg)	89	21
			Dry swallowing pressure in the velopharyngeal muscle zone (mmHg)	95	96
			UES Zone (mmHg)	171	75

*Cm*, centimetre; *FOIS*, functional oral intake scale; *mmHg*, unit of pressure; *P1*, participant 1; *P2*: participant 2; *s*: second

### Change to Bolus Characteristics

#### Sensory Stimulation

One out of the two PwMND in Regan’s [26] study could not tolerate sensory stimulation. Data from the remaining participant are reported in Table 4. Cold, sour and carbonated boluses caused a considerable increase on the velopharyngeal mesopharyngeal and global pharyngeal contractile vigour. Sensory stimulation did not alter the hypopharyngeal contractile integral as significantly, except the carbonated bolus, which increased this measure.

#### Altering Bolus Consistency

Normal saline liquid and viscous boluses were trialled with PwMND in Cock and Colleagues’ [25] study. The addition of viscosity resulted in a reduction in pharyngeal peak pressure in PwMND. Effects of the viscous bolus on the swallow of PwMND can be found in Table 4.

### Manoeuvres

#### Effortful Swallow

Both PwMND included in Heslin’s [27] study tolerated and completed the effortful swallow manoeuvre. UES relaxation duration was increased in both participants when the manoeuvre was applied. Further effects of such are outlined in Table 4.

### Surgical

#### Cricopharyngeal Myotomy

Takasaki et al. [23] evaluated the swallowing pressure in a patient with MND one month before and three months after bilateral cricopharyngeal myotomy. As outlined in Table 4, the patient’s velopharyngeal pressures did not change after surgery. The values of the UES, on the other hand, decreased significantly.

## Discussion

This study sought to explore the use of PHRM to evaluate dysphagia in PwMND. The inclusion of six publications, half of which contained a sample size of two or less participants, suggests that the current use of PHRM to evaluate dysphagia in PwMND is quite limited. This is despite the fact there has been a recent shift towards proactive dysphagia management in PwMND to optimise physiological reserve. As PHRM is being adopted into clinical practice internationally [32] the use of PHRM to evaluate dysphagia in this clinical population is likely to increase in the future.

Several important findings based on this limited data are highlighted in this review. This scoping review has demonstrated considerable variability in PHRM protocol and analysis methods in PwMND. There was variability in HRM systems used, HRM catheter dimensions, bolus volumes and consistencies administered and PHRM metrics obtained. Each of these variables can alter the pressure measurements obtained, limiting comparison of study findings [18]. Winiker et al. [31] reported considerable variability in PHRM protocol and gaps in documentation of such protocol across the literature. This review reveals that studies using PHRM in PwMND are no exception to these gaps or discrepancies. The combination of the variation in PHRM protocol and analysis methods across research centres and PHRM pressure variability with age, volitional swallow tasks and pharyngeal region previously described [13, 14] have the potential to markedly impact on clinical judgement in PHRM evaluation and warrant considerable caution until our understanding of PHRM best practice evolves. Pressure variability is of particular relevance to PwMND as this population is likely to present with impaired swallow motor control and an unpredictable swallow performance [14].

PHRM is an emerging technology and standardised guidelines for protocol and analysis methods have yet to be fully established. The PHRM international working group published the first set of protocol and metric recommendations which may streamline PHRM protocols in future dysphagia research [18]. A 5-min accommodation period, bolus delivery via syringe and the use of a solid-state HRM system with at least 10 pressure sensors 1 cm apart is advised. The magnitude of variability in the PHRM protocol and analysis methods and gaps in the documentation of such across the included studies is likely due to the fact that four out of six of the studies were published before these recommendations. One of the remaining three studies is an abstract only [28], thus justifying its lack of documentation. It is encouraging to note that full compliance to the recommendations was noted in the outstanding studies [26, 27], suggesting that the most recent publications are adhering to these preliminary guidelines. Continued compliance to the working groups’ recommendations in future publications will increase comparability of results across papers and will, in turn, enhance and further improve the understanding of the nature and course of dysphagia in PwMND.

From a PHRM feasibility perspective, PwMND across all FOIS levels in all studies that documented outcomes completed the PHRM assessment. Furthermore, the studies that reported adverse events reported that no side effects or adverse events occurred. This finding suggests that despite bulbar dysfunction in MND, PHRM is a feasible tool for this clinical population. This finding has somewhat been reflected in the previous literature. Transnasal endoscopic procedures pose similar risks to the transnasal passage of the PHRM catheter [33]. Studies documenting such procedures in PwMND have reported a high-completion rate and low incidence of adverse events [34, 35]. The advancement of the PHRM catheter through the UES, which is not seen in FEES, introduces additional considerations [33]. Whilst this is the first study to outline the feasibility of PHRM in PwMND, previous studies have documented a high-completion rate of PHRM in people with dysphagia, concluding that it is a safe and practical assessment [33]. It is of interest, however, that only one study documented patient tolerability and none of the included studies documented patient-reported outcomes of the assessment. This is a striking finding and researchers should be aware that data regarding patient tolerability are integral for an assessment that is translating into clinical practice [31]. Capturing the patients’ experience holds increased importance for this clinical population as features of MND such as hyperactive gag reflex and weak cough may impact patient acceptability significantly [6, 7]. Thus, whilst the limited data obtained suggest that PHRM is a feasible tool, gaps in documentation of patients’ experience obscures the certainty of this finding.

PHRM was used across studies to provide quantitative novel data on the velopharynx, mesopharynx, hypopharynx and the UES in PwMND. The aforementioned variability in protocol and analysis methods restricts the comparability of the metric results across the studies. Nevertheless, preliminary data published to date suggest that alterations in swallow pressure are present, and they can be identified in the velopharynx, mesopharynx and hypopharynx during swallowing. PHRM, therefore, has the potential to provide clinically useful quantitative data on swallow pathophysiology that cannot be captured through VFS or FEES. This data has the potential to inform proactive dysphagia intervention in this population to maximise physiological reserve. Of note, within-subject pressure variability has been identified across pharyngeal regions (and most notably the hypopharyngeal region) and this needs to be considered in the context of these findings [14].

When compared against normative data, the most dominant changes in swallowing physiology in PwMND highlighted through PHRM included (i) reduced pressure and contractility in the mesopharyngeal, hypopharyngeal and global pharyngeal region and (ii) evidence of UES restriction. These findings somewhat align with the previous literature as ‘changes in muscle tone’ and ‘reduced constriction’ have been previously reported [1, 7, 8]. However, this comparison further highlights that PHRM provides enhanced insight into the swallowing physiology in PwMND, as regional detail on pressure changes and exact, reliable findings of the UES have not been summarised in the literature before. Whilst these findings are based on limited data and further research is required to delineate the nature of these results, it can be concluded that PHRM offers an enhanced, comprehensive and specific insight into the changes of the swallow in PwMND.

This review highlights the potential role of PHRM to determine the discrete benefits of dysphagia interventions on swallowing in PwMND. To date, PHRM has been used in four studies to delineate specific effects of surgical and compensatory dysphagia interventions, including cricopharyngeal myotomy, sensory stimulation, increased bolus consistency and effortful swallow. The impact of cricopharyngeal myotomy on PwMND has been previously documented in a dated VFS study; reported findings were vague as the procedure was documented to ‘improve swallow function’ [36]. This review highlights that PHRM provides a much more specific and objective account of the effects of the intervention. The quantitative effects of the cricopharyngeal myotomy on the UES and velopharyngeal regions in the participant with MND were provided in the included PHRM study, rather than a descriptive subjective claim of improvement. The impact of thickened liquids has been reported to reduce pharyngeal constriction in PwMND in a VFS study [37]. These findings somewhat align with the results of the included PHRM study as reduced pharyngeal pressures were reported in response to increased bolus consistency in PwMND [25]. The PHRM study, unlike the VFS study, provided an insight into the quantitative degree of reduction, enabling a more comprehensive and definite insight into the effects of the intervention. From an effortful swallow perspective, recent research has determined that volitional swallow tasks can increase within-subject pressure variability [14]. This increased pressure variability is purported to be due to the increased task complexity and reduced automaticity of the volitional task compared to baseline swallow [14]. This issue needs to be considered when designing future PHRM study protocols. Further research into the effects of proactive dysphagia interventions on PwMND as measured by PHRM would increase clinician and researchers understanding of the nature and impact of dysphagia interventions and would ensure that only the most beneficial interventions are applied.

The findings of this review carry clinical significance as they inform clinicians of the value and viability of completing the PHRM assessment with PwMND. This review suggests that PHRM is a feasible tool for PwMND that can be utilised by clinicians to obtain a specific insight into the biomechanics of the swallow as well as an overview of the discrete and subtle effects of dysphagia interventions.

This scoping review of the adoption of PHRM into dysphagia evaluation in PwMND is the first of its kind and serves as a basis for guiding future research in this field. In order to enhance the understanding of the use of PHRM in PwMND, future research should focus on the feasibility of PHRM in this clinical population, highlighting patient acceptability and patient-reported outcomes of the assessment. Researchers should adhere to the PHRM international working groups’ recommendations and follow the protocol and select the metrics that are advised. Given the small sample sizes across all included studies, it is recommended that researchers come together and collaborate to conduct large-scaled multi-site research. This would increase the sample size, the quality of the research and in turn our understanding of the use of PHRM to evaluate dysphagia in PwMND.

### Conclusion

Few studies have reported the use of PHRM in PwMND, thus it can ultimately be concluded that the current understanding of the adoption of PHRM into dysphagia evaluation in PwMND is limited. Whilst variability in PHRM protocol and analysis methods in PwMND restricts the comparability of the metric results, PHRM appears to be a feasible tool for this clinical population. PHRM can provide novel data on the swallow biomechanics in PwMND, offering an enhancing and detailed insight into the subtle and specific physiological changes in the swallow that occur in PwMND. Additionally, given the recent move from compensatory dysphagia management in this population, PHRM may identify therapeutics targets and quantify benefits to proactive rehabilitation. Further research is required to advance the understanding of the adoption of PHRM into dysphagia evaluation in PwMND.

## Supplementary Information

Below is the link to the electronic supplementary material.

Supplementary file1 (DOCX 462 KB)
