# Supplementary material for: Use of Pharyngeal High-Resolution Manometry to Evaluate Dysphagia in Adults with Motor Neurone Disease: A Scoping Review
Source: Dysphagia. 2022 Mar 2;37(6):1697–714. doi: 10.1007/s00455-022-10418-4 (PMC9643180; doi:10.1007/s00455-022-10418-4)

**Appendices:**

***Database search strings.***

PubMed Search: *In text: 35 results*

CINAHL search:

( TI ( deglutition* OR swallow* OR oropharyngeal OR pharyn* OR dysphagi* OR feed* OR fed OR eat OR eating OR eats OR ate OR drink* OR drank ) OR AB ( deglutition* OR swallow* OR oropharyngeal OR pharyn* OR dysphagi* OR feed* OR fed OR eat OR eating OR eats OR ate OR drink* OR drank ) OR (MH "Deglutition") OR (MH "Deglutition Disorders") ) AND ( TI ( “motor neurone disease*” OR “motor neuron disease*” OR MND OR ALS OR “motor system disease*” OR “anterior horn cell disease*” OR “lateral sclerosis” OR “lateral scleroses” OR “progressive bulbar palsy” OR “bulbar paralysis” OR “progressive muscular atroph*” OR “charcot disease*” OR “Lou Gehrig” OR “mixed etiolog*” OR “mixed aetiolog*” ) OR AB ( “motor neurone disease*” OR “motor neuron disease*” OR MND OR ALS OR “motor system disease*” OR “anterior horn cell disease*” OR “lateral sclerosis” OR “lateral scleroses” OR “progressive bulbar palsy” OR “bulbar paralysis” OR “progressive muscular atroph*” OR “charcot disease*” OR “Lou Gehrig” OR “mixed etiolog*” OR “mixed aetiolog*” ) OR (MH "Motor Neuron Diseases") ) AND ( TI ( manometry OR high resolution OR “pharyngeal pressure*” ) OR AB ( manometry OR high resolution OR “pharyngeal pressure*” ) OR (MH "Manometry") )

*Limiters: Exclude Medline records: 1 result*

EMBASE search:

(**deglutition***:ab,ti OR **swallow***:ab,ti OR **oropharyngeal**:ab,ti OR **pharyn***:ab,ti OR **dysphagi***:ab,ti OR **feed***:ab,ti OR **fed**:ab,ti OR **eat**:ab,ti OR **eating**:ab,ti OR **eats**:ab,ti OR **ate**:ab,ti OR **drink***:ab,ti OR **drank**:ab,ti OR **'swallowing'**/exp OR **'dysphagia'**/exp) AND (**'motor neurone disease*'**:ab,ti OR **'motor neuron disease*'**:ab,ti OR **mnd**:ab,ti OR **als**:ab,ti OR **'motor system disease*'**:ab,ti OR **'anterior horn cell disease*'**:ab,ti OR **'lateral sclerosis'**:ab,ti OR **'lateral scleroses'**:ab,ti OR **'progressive bulbar palsy'**:ab,ti OR **'bulbar paralysis'**:ab,ti OR **'progressive muscular atroph*'**:ab,ti OR **'charcot disease*'**:ab,ti OR **'lou gehrig'**:ab,ti OR **'mixed etiolog*'**:ab,ti OR **'mixed aetiolog*'**:ab,ti OR **'motor neuron disease'**/exp) AND (**manometry**:ab,ti OR **'high resolution'**:ab,ti OR **'pharyngeal pressure*'**:ab,ti OR **'manometry'**/exp)

*Limiter: Exclude Medline record: 22 results*

Web Of Science Core search:

***TOPIC:****(deglutition* OR swallow* OR oropharyngeal OR pharyn* OR dysphagi* OR feed* OR fed OR eat OR eating OR eats OR ate OR drink* OR drank) AND****TOPIC:****(“motor neurone disease*” OR “motor neuron disease*” OR MND OR ALS OR “motor system disease*” OR “anterior horn cell disease*” OR “lateral sclerosis” OR “lateral scleroses” OR “progressive bulbar palsy” OR “bulbar paralysis” OR “progressive muscular atroph*” OR “charcot disease*” OR “Lou Gehrig” OR “mixed etiolog*” OR “mixed aetiolog*”) AND****TOPIC:****(manometry OR high resolution OR “pharyngeal pressure*”)*

*No Limits: 56 results*


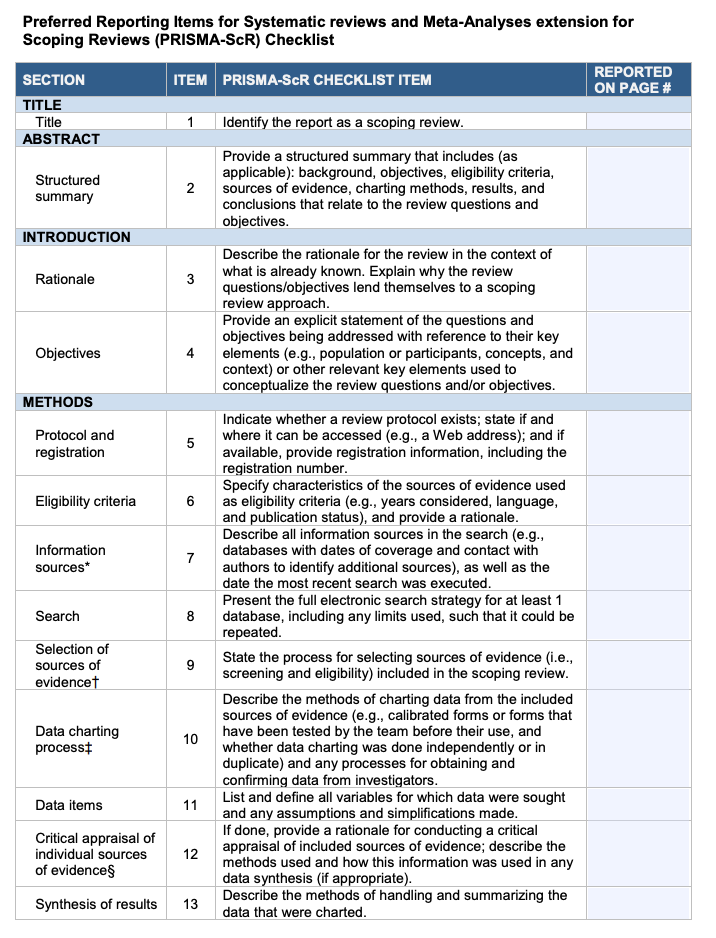


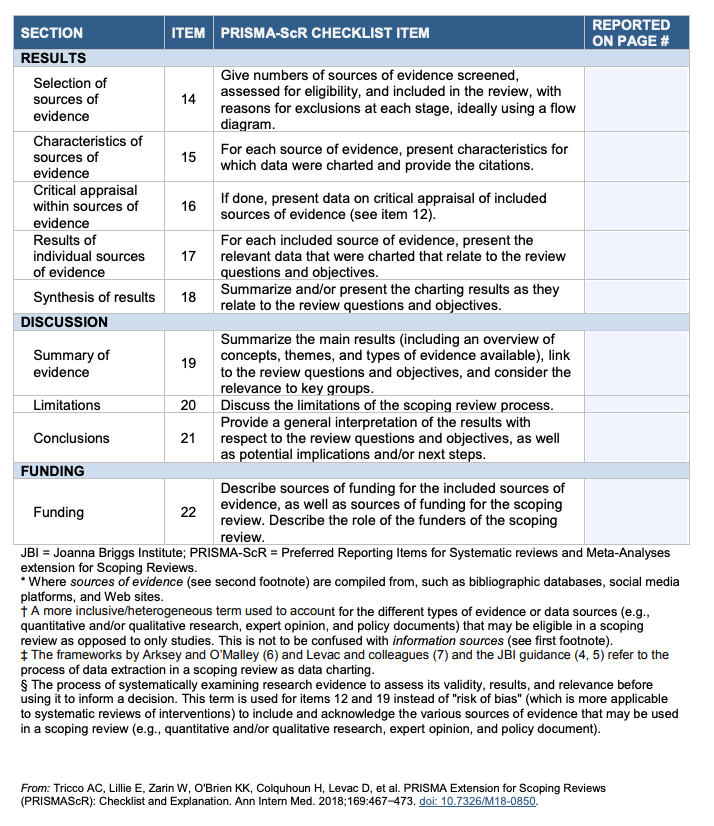

Supplement: Supplementary file 1 — Supplementary file1 (DOCX 462 KB) [file 455_2022_10418_MOESM1_ESM.docx]
